# Human parainfluenza virus in patients with influenza-like illness from Central and South America during 2006–2010

**DOI:** 10.1111/irv.12211

**Published:** 2013-11-28

**Authors:** Manuel V Villaran, Josefina García, Jorge Gomez, Ana E Arango, Marina Gonzales, Wilson Chicaiza, Washington Alemán, Ivette Lorenzana de Rivera, Felix Sanchez, Nicolas Aguayo, Tadeusz J Kochel, Eric S Halsey

**Affiliations:** a Virology, U.S. Naval Medical Research Unit SixLima, Peru; b Direccion General de Epidemiologia, Ministerio de SaludLima, Peru; c Universidad de AntioquiaMedellin, Colombia; d Laboratorio Departamental, Secretaria Seccional de Salud del MetaVillavicencio, Colombia; e Hospital Vozandes, Universidad de las AmericasQuito, Ecuador; f Clinica Alcivar, Hospital VernazaGuayaquil, Ecuador; g Universidad Nacional Autonoma de HondurasTegucigalpa, Honduras; h Hospital Infantil Manuel de Jesus RiveraManagua, Nicaragua; i ONG Rayos de SolAsunción, Paraguay

**Keywords:** Epidemiology, Latin America, parainfluenza virus, phylogenetics

## Abstract

**Background:**

Human parainfluenza viruses (HPIVs) are common viral causes of community-acquired pneumonia, particularly in children. The four types of HPIV have world-wide distribution; however, limited information exists about the epidemiological profile of HPIV in Latin-America.

**Objective:**

Provide epidemiologic and phylogenetic information about HPIVs that circulated in Latin America between 2006 and 2010 to better characterize the extent and variability of this respiratory virus in the region.

**Methods:**

Oropharyngeal swabs, demographic data and clinical characteristics were obtained from individuals with influenza-like illness in 10 Latin-American countries between 2006–2010. Specimens were analyzed with culture and molecular methods.

**Results:**

A total of 30 561 individuals were enrolled; 991 (3·2%) were HPIV positive. Most infected participants were male (53·7%) and under 5 years of age (68·7%). The HPIV type most frequently isolated was HPIV-3 (403, 40·7%). In 66/2007 (3·3%) hospitalized individuals, HPIV was identified. The most frequent symptoms at enrollment were cough and rhinorrhea. We identified certain patterns for HPIV-1, -2 and -3 in specific cities. Phylogenetic analysis revealed a homogeneous distribution in the region.

**Conclusions:**

In the current scenario, no vaccine or treatment is available for this pathogen. Our results contribute to the scarce epidemiologic and phylogenetic information of HPIV in the region that could support the development of specific management.

## Background

Human parainfluenza viruses (HPIVs) were first described in the 1950s,^1^ and although resembling myxoviruses (e.g., influenza virus), their poor growth in embryonated eggs and different antigenic sites placed them into a new family of viruses, *Paramyxoviridae* (*Respivirus* genus). HPIVs are enveloped non-segmented, negative, single-stranded RNA viruses. Four types of HPIVs exist, each with different genetic and antigenic characteristics.^1^,^2^ These respiratory tract pathogens can infect individuals of any age group. HPIVs are a common viral cause of community-acquired pneumonia in healthy adults,^3^ but also are one of the most common viral causes of pediatric hospitalizations due to respiratory disease, accounting for nearly one-third of lower respiratory infections in children under 5 years.^4^ Immune-related factors (e.g., bone marrow transplantation) and virus-related factors (e.g., type 3) have been linked to worse outcomes and outbreaks.^1^,^5^–^8^

Overcrowding, malnutrition, lack of breastfeeding, and air pollution have been associated with HPIV infection, regardless of severity.^9^ Limited information exists about the epidemiological profile of HPIV in Latin America, and most of it is in the pediatric population of countries such as Colombia, Argentina, Mexico, and Brazil.^10^–^15^

The Naval Medical Research Unit No.6 (NAMRU-6) has conducted surveillance of viral respiratory pathogens in several Latin American countries over the past decade.^16^–^18^ Presently, no vaccine or treatment is available for this pathogen. The objective of this study was to provide epidemiologic and phylogenetic information about HPIVs that circulated in Latin America between 2006 and 2010 to better characterize the extent and variability of this respiratory virus in the region.

## Methods

### Ethics

The data presented in this article were obtained as part of the surveillance protocol “Surveillance for Emerging Viral Respiratory Pathogens in South America – NMRCD.2002.0019,” approved as less than minimal risk by the Naval Medical Research Center Detachment (NMRCD, now known as Naval Medical Research Unit No.6 (NAMRU-6)) Institutional Review Board (IRB), the Bioethical Committee of the Universidad Central de Ecuador, the Sociedad Ecuatoriana de Bioetica, and the ministries of health of all the participant countries. Databases were shared with local authorities, such as ministries of health, as accorded in each country.

### Case definition

Influenza-like illness (ILI) was defined as a sudden onset of fever (≥38°C) and cough or sore throat fewer than 5 days in duration.^19^ Fever was obtained orally or rectally in the healthcare center. Subjects without fever in the clinic (e.g., secondary to taking antipyretics) were also recruited if they had a history of a measured temperature ≥38°C or greater at home. At each site, trained medical personnel were responsible for properly identifying and classifying cases.

### Study population

Samples and data were obtained from participants in Honduras, Nicaragua, El Salvador, Venezuela, Colombia, Ecuador, Peru, Bolivia, Paraguay, and Argentina. The study population included every individual, regardless of age, who sought attention at any of the 49 outpatient health facilities participating in the surveillance between June 2006 and November 2010, fulfilled the case definition and agreed to participate in the study.

**Figure 1:**
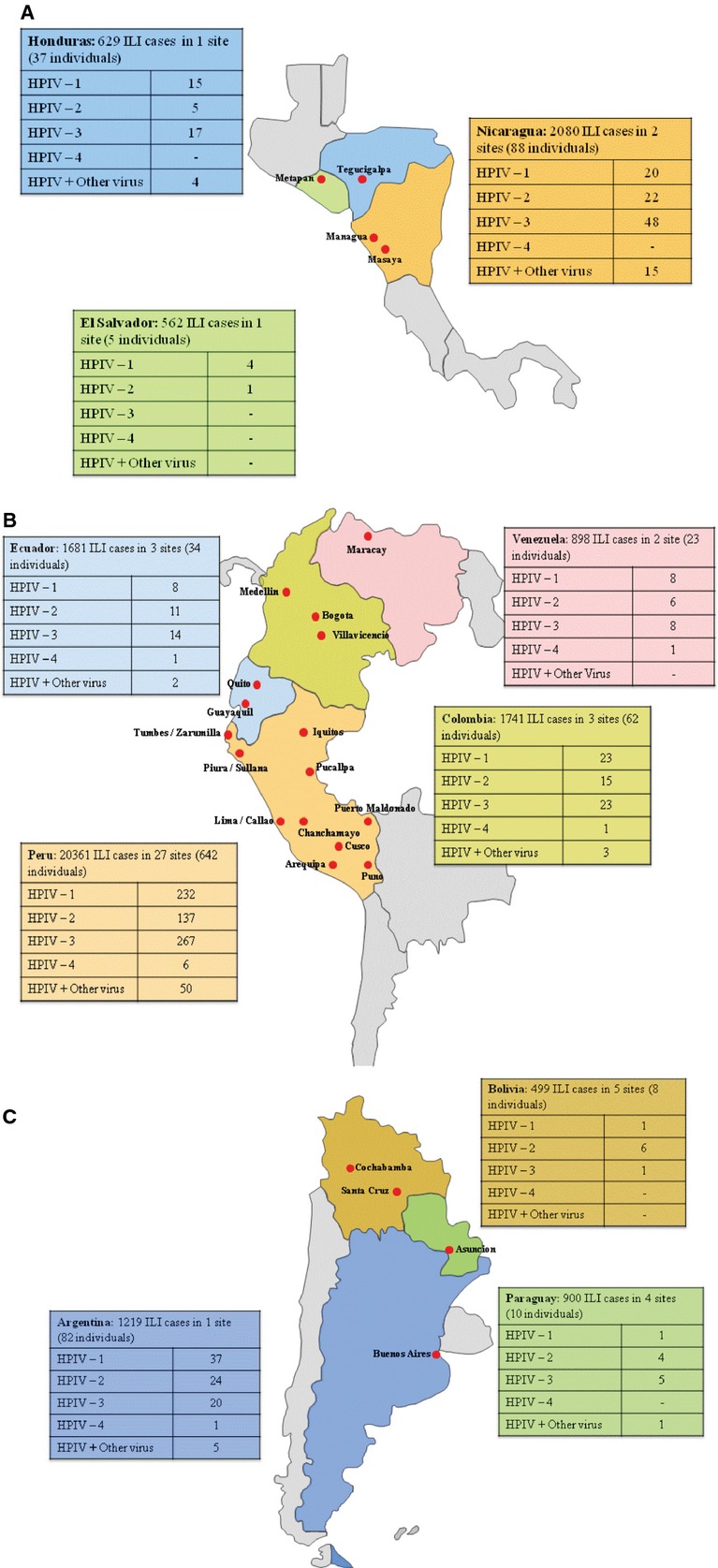
Country distribution of HPIV, June 2006 – November 2010. (A) Participating cities in Central America; (B) Participating cities in the northern area of South America; (C) Participating cities in the southern area of South America.

The 24 cities participating (Figure 1) covered a wide spectrum of climates and regions, including coastal (Lima, Peru, and Guayaquil, Ecuador), highland (Bogota, Colombia, and Quito, Ecuador), and rainforest (Managua, Nicaragua, and Iquitos, Peru).

### Data and specimens collection

Demographic data, risk factors, and clinical characteristics were recorded using a standardized case report form.

Oropharyngeal swabs were collected at all sites except in Nicaragua where nasopharyngeal swabs were collected for viral isolation. Swabs were placed in transport media and stored at −70°C until they were delivered on dry ice to NAMRU-6 in Lima, Peru.

### Virus isolation and identification

Three cell lines for virus isolation were used in this study: Madin-Darby canine kidney (MDCK), African green monkey kidney (VeroE6), and Rhesus monkey kidney (LLCMK2) cells. Each cell line was prepared in 24-well tissue culture plates. The growth medium for the three cell lines consisted of Eagle's minimum essential medium (Quality Biological, Cat.112-018-131) with 10% fetal bovine serum (F-4135, Sigma-Aldrich, St. Louis, MO, USA) and antibiotic–antimycotic solution (10 000 units penicillin, 10 mg streptomycin and 25 μg amphotericin B) (A-5955, Sigma). The patients' specimens were also treated with the antibiotic/antimycotic solution to inhibit flora.^20^

For 10 days (13 days in the case of Vero cells), cell cultures were examined daily by microscope to detect cytopathic effect, which was considered present if focal rounding and destruction, elongated cells, and/or occasional syncytium formation were found. Either upon observation of cytopathic effect or after 10 days (13 in the case of Vero cells), cell suspensions were dried and fixed in chilled acetone for 15 minutes. Immunofluorescence assay (IFA) was performed to identify the virus isolates using a direct fluorescence assay. The Respiratory Virus Screening and Identification Kit (D3 DFA Respiratory Virus Diagnostic Hybrids; Athens, OH, USA) was utilized for the identification of HPIV-1, HPIV-2, and HPIV-3, adenovirus, influenza A virus, influenza B virus, and RSV. The anti-parainfluenza 4 antibody, clone 531-1G (Merck Millipore; Billerica, MA, USA), was utilized for the identification of HPIV-4 (starting in 2009). The D3 DFA HSV identification kit and D3 IFA Enterovirus ID Kit (Diagnostic Hybrids) were used to identify herpes simplex virus and enteroviruses, respectively. All assays were performed following the manufacturers' established protocols. In addition to culture, all samples underwent reverse-transcription PCR to detect influenza viruses, as this study was nested in a larger influenza virus surveillance project.^17^

Ten percent of each HPIV type identified as positive by culture and IFA was randomly chosen for evaluation by real time polymerase chain reaction (RT-PCR) and sequencing. Randomization was carried out using Stata/SE 10.0 for Windows (StataCorp LP; College Station, TX, USA). RNA was extracted using the QIAamp Viral RNA Mini spin protocol (Qiagen; Hilden, Germany). Viral RNA extraction was performed in a biosafety level-3 laboratory with enhanced containment practices. Nucleic acid was extracted from 140 μl of the pharyngeal swabs with the use of a viral RNA kit (QIAamp, Qiagen), re-suspended in 60 μl of buffer AVE, and amplified.

The primers used for RT-PCR and sequencing were specific to the hemagglutinin-neuraminidase (HN) gene segments of HPIV-1, HPIV-2, and HPIV-3^21^; for HPIV-4, the primers were specific to the phosphoprotein (P) gene.^22^ The primers used were as follows:

HVP1: PIP1 + (5′-CCT TAA ATT CAG ATA TGT AT-3′), PIP1- (5′-CGT ATC AAT AAT TAT TTA TC-3′) 477 bp;
HVP2: PIP2 + (5′-AAC AAT CTG CTG CAG CAT TT-3′), PIP2- (5′-ATG TCA GAC AAT GGG CAA AT-3′) 507 bp;
HVP3: PIP3 + (5′-CTG TAA ACT CAG ACT TGG TA-3′), PIP3- (5′-TTT AAG CCC TTG TCA ACA AC-3′) 477 bp;
HVP4: PIP4 + (5′-CTGAACGGTTGCATTCAGGT-3′), PIP4- (5′-AGG ACT CAT TCT TGA TGC AA-3′) 441 bp.

RT-PCR was performed in 24 μl of reaction mixture consisting of 3 μl of nuclease-free water, 2·5 μl of MgSO_4_, 12·5 μl of 2× reaction mix (Super Script III One- Step RT-PCR System with platinum Taq High Fidelity kit), 0·5 μl of 20 μm concentrations of sense and antisense primers, 0·5 μl of enzyme mix, and 5 μl of template. Cycling conditions were 30 minutes at 50°C, 2 minutes at 94°C, followed by 40 cycles of 30 seconds at 94°C, 120 seconds at 50°C, 120 seconds at 72°C, and finishing with a 7 minute hold at 72°C. The PCR products were sized by gel electrophoresis on 2% agarose and then visualized under UV light. The products were purified with Centri-Sep columns prior to cycle sequencing using the Dye Terminator Big Dye v3.1 Kit (Applied Biosystems; Foster City, CA, USA). The analysis was carried out in an ABI 3130xl Genetic Analyzer (Applied Biosystems).

### Sequencing and phylogenetic analysis

Nucleotides of the HN or P protein genes were amplified, sequenced, and compared to sequences from GenBank. Nucleotide sequences were aligned using Clustal X. Phylogenetic analyses were performed using the Kimura two-parameter model as a model of nucleotide substitution and using the neighbor-joining method to reconstruct phylogenetic trees MEGA version 2.1, developed by (Sudhir Kumar, Koichiro Tamura, Ingrid B. Jakobsen, and Masatoshi Nei; MEGA2: molecular evolutionary genetics analysis software, Arizona State University, Tempe, Arizona). We labeled the samples according to the following format: “Country of collection – Sample code – Month – Year of collection.” The comparison sequences were labeled according to the following format: “Human parainfluenza virus – Country of collection – Year of collection – Accession Number.”

### Statistical analysis

The data obtained using the case report form were entered into a database using Microsoft Access and analyzed using Stata/SE 10.0 for Windows (StataCorp LP). Chi-square and Fisher tests were used to assess associations. Two-proportion z-test was used to compare proportions; *P*-values ≤0·05 were considered statistically significant. Annual HPIV activity, including primary peaks, were assessed using EPIPOI (Analytical Software for Epidemiological Time Series 23).

## Results

Between June 2006 and November 2010, a total of 30 561 respiratory samples were obtained in the 10 participating countries. Of these, 991 (3·2%) were positive for HPIV by either isolation or PCR. The distribution of the positive samples among the countries is shown in Figure 1.

Most participants infected with HPIV were male (532, 53·7%) and under 5 years of age (681/991, 68·7%), ranging between 0 and 73 years with a median age of 2 (interquartile range [IQR] 4; Table 1). Although we noted no association between gender and isolation of HPIV, we did find a fourfold increased chance of HPIV isolation in those 5 years old or younger (OR = 4·4, 95% CI 3·9 – 5·1; *P*-value <0·01) compared with those older than 5 years. When assessed individually, HPIV-1, HPIV-2, and HPIV-3, all had a higher probability of isolation in this younger age group (OR = 4·5, 95% CI 3·60 – 5·89, *P*-value <0·01; OR = 2·24, 95% CI 1·68 – 2·98, *P*-value <0·01, and OR = 6·61, 95% CI 5·15 – 8·56, *P*-value <0·01, respectively). However, the proportion of HPIV per total ILI cases in children 5 years old or younger compared with the rest of the study population was not statistically significant.

**Table 1:** Characteristics of the study population

	Surveyed individuals no. (%)	Individuals with HPIV no. (%)
Number of individuals	30 561	991 (100)
Demographic data		
Sex*		
Female	14 924 (48·8)	459 (46·3)
Male	15 631 (51·2)	532 (53·7)
Age in years**		
Mean ± SD	16·3 ± 17·1	6·9 ± 11·9
Median [Range]	10 [0–100]	2 [0–73]
Q_25_–Q_75_	2–25	1–5
0–5	10 579 (34·6)	678 (68·6)
6–14	6991 (22·9)	176 (17·8)
15–29	6954 (22·8)	68 (6·9)
30–44	3329 (10·9)	34 (3·4)
45–59	1739 (5·7)	20 (2·0)
≥60	964 (3·1)	12 (1·2)
Isolation***		
HPIV-1		349 (35·2)
HPIV-2		231 (23·3)
HPIV-3		403 (40·7)
HPIV-4		10 (1·0)
HPIV + other virus†		80 (–)

* Not all surveyed individuals provided their gender.

** In the surveyed population, 30 556 provided age information; in the HPIV-positive population, 998 provided age information.

*** Total exceeds 100·0% because more than one HPIV was isolated from two samples.

† Including more than one HPIV.

The type of HPIV most frequently isolated was HPIV-3 (403, 40·7%). In 80 samples, one or more viruses were isolated in addition to HPIV. The most frequent viruses isolated with HPIV were (Table 2) influenza A virus (31, 39%), adenovirus (17, 21%), and herpes simplex virus (12, 15%). Other viruses found with HPIV were influenza B virus, RSV, and enteroviruses (including coxsackievirus). We also noted the presence of two different HPIVs in two samples.

**Table 2:** Viruses isolated with HPIV

Virus	Frequency (%)
Influenza A virus	31 (39)
Adenovirus	16 (20)
Herpes simplex virus	12 (15)
Enterovirus (including Coxsackie virus)	9 (11)
Influenza B virus	5 (6)
RSV	3 (4)
HPIV-1 and HPIV-3	1 (1)
HPIV + more than one virus*	3 (4)

* HPIV and: Adenovirus – Enterovirus; Adenovirus – Herpes Simplex Virus; Influenza B – HPIV-2 – HPIV-3.

Of the 30 561 patients recruited in this outpatient study, a total of 2007 were subsequently hospitalized on the enrollment day; in 66 of those, HPIV was isolated (median age 1, IQR 2·2). Fifty-four (82%) of those hospitalized with HPIV infection were ≤5 years old. The HPIV type most frequently isolated in the hospitalized participants, regardless of age, was HPIV-3 (33, 50%; Table 3). Although no association was found between HPIV-1, HPIV-2, and HPIV-4 and patient hospitalization, a significant association was found with HPIV-3 (χ^2^ = 4·79; *P*-value = 0·03).

**Table 3:** HPIV isolated in hospitalized participants by age

Culture	Total (%)	<1 year (%)	1–5 years (%)	6–12 years (%)	13–18 years (%)	19–59 years (%)	≥60 years (%)
HPIV-1	18 (27)	5 (19)	9 (32)	1 (25)	0 (0)	3 (60)	0 (0·00)
HPIV-2	9 (14)	2 (8)	4 (14)	2 (50)	1 (100)	0 (0)	0 (0)
HPIV-3	33 (50)	18 (69)	12 (43)	0 (0)	0 (0)	2 (40)	1 (50)
HPIV-4	1 (2)	1 (4)	0 (0)	0 (0)	0 (0)	0 (0)	0 (0)
HPIV + other virus*	5 (7)	0 (0)	3 (11)	1 (25)	0 (0)	0 (0)	1 (50)
Total	66 (100)	26 (100)	28 (100)	4 (100)	1 (100)	5 (100)	2 (100)

* Including more than one HPIV.

The most frequent symptoms at enrollment (including those who got hospitalized) were cough (an inclusion criterion) and rhinorrhea. However, a frequent symptom among hospitalized participants was respiratory difficulty (37, 56%). Two participants developed multi-organ failure: a 6-month-old male child and a 71-year-old man. HPIV-2 was isolated in the child, and HPIV-3 and herpes simplex virus were isolated in the adult.

HPIV 1-3 were present in the region throughout the 54 months of surveillance of this study, with HPIV-1 being the predominant HPIV type in Argentina, El Salvador, and Venezuela; HPIV-2 being the predominant HPIV type in Bolivia and Paraguay; and HPIV-3 being the predominant HPIV type in Colombia, Ecuador, Honduras, Nicaragua, Paraguay, Peru, and Venezuela (we found equal number of isolations of HPIV-1 and HPIV-3 in Venezuela, and HPIV-2 and HPIV-3 in Paraguay). Assays for HPIV-4 were introduced in late 2009, and the virus was isolated initially in 2010. Therefore, only 8 HPIV-4 isolates were identified during the study period, and this type was excluded from temporal and geographic analyses.

**Figure 2:**
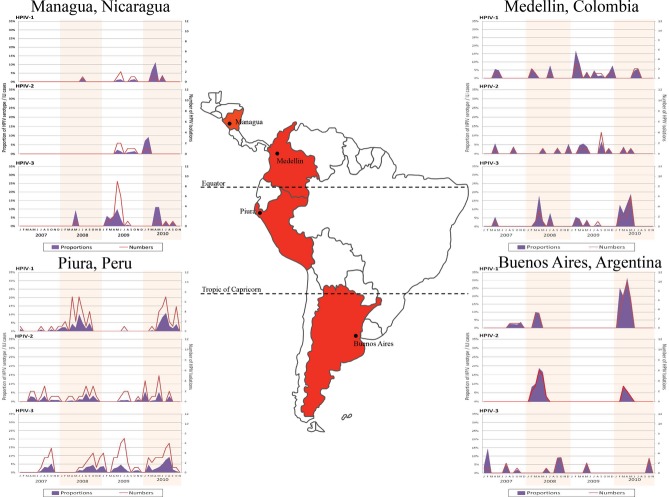
Annual HPIV activity.

During the study period, when pooled regardless of the site of origin, 35% of the HPIV isolations occurred between the months of June and August. We selected four cities with steady enrollment during the study period and different latitudes in Central and South America (Figure 2) to show HPIV-1, HPIV-2, and HPIV-3 behavior within the region between 2007 and 2010 (2006 was not included because surveillance was not yet consistent). In Managua, Nicaragua (latitude 12°8′11″ N), irregular patterns for HPIV-1 and HPIV-2 were found with an apparent primary peak around June; however, HPIV-3 was consistently responsible for spikes of ILI during the first semester of years 2007–2010, presenting a primary peak between May and June; Medellin, Colombia (latitude 6°14′9″ N), also presented irregular patterns for HPIV-1 and HPIV-2. As in Managua, Medellin had a disproportionate number of HPIV-3 isolations in the first halves of the years 2008–2010. An annual pattern depicts an increased number of HPIV-3 cases during the first semester of each analyzed year. Piura, Peru (latitude 5°12′0″ S), had similar proportions of HPIV-1 and HPIV-2 (including an almost absence of these pathogens during 2009); however, the primary peaks for HPIV-1 occurred between June and July, while for HPIV-2, around August; HPIV-3 had an irregular presentation during the study period. Nevertheless, the primary peak was calculated to be between June and July, also. Finally, in Buenos Aires, Argentina (latitude 34°36′12″ S), HPIV-1 and HPIV-2 behaved in a similar fashion, with biannual peaks occurring between April and May and an apparent absence during 2009; HPIV-3 presented its primary peak around October with no further identifiable pattern.

**Figure 3:**
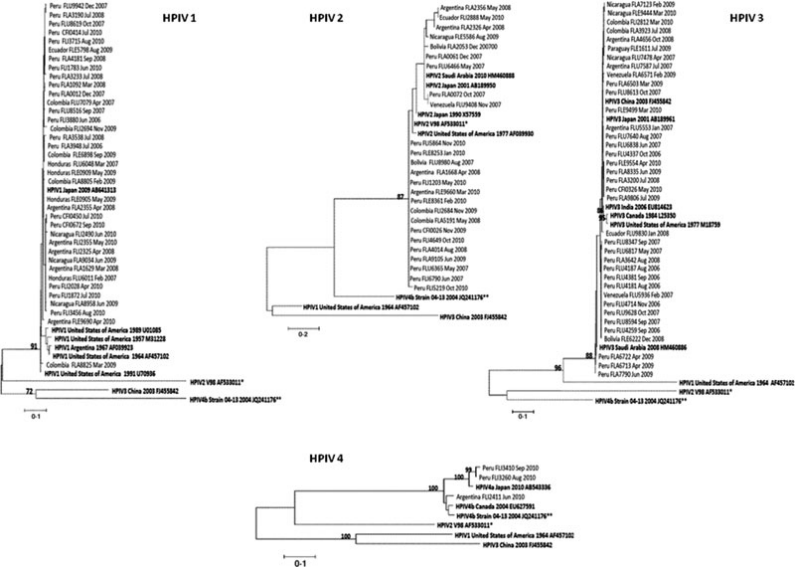
Phylogenetic analysis. Nucleotides of the HN protein gene of HPIV-1, -2, and -3 and of the P protein gene of HPIV-4 were amplified, sequenced, and compared to published sequences from GenBank. We labeled the samples according to the following format: “Country of collection - Sample code - Month - Year of collection.” The comparison sequences were labeled according to the following format: “Human parainfluenza virus – Country of collection – Year of collection – Accession Number”. Nucleotide sequences were aligned using Clustal X. Phylogenetic analyses were performed using the Kimura two-parameter model as a model of nucleotide substitution and using the neighbor-joining method to reconstruct phylogenetic trees (MEGA version 2.1). *Strain V98's country and year of collection was not available in GenBank. ** Strain 04-13's country of collection was not available in GenBank.

Our phylogenetic analyses (Figure 3) illustrate the great variety of circulating HPIV viruses in the southern hemisphere. HPIV-1 was distributed throughout Latin America, with the majority related to a 2009 strain from Yamagata, Japan (AB641313**)**, and, to a lesser extent, to older strains detected in 1957 and 1989 in the USA (M31228 and U01085). HPIV-2 showed more nucleotide variability than the other HPIV types, although this did not translate into more amino acid variability. All of our HPIV-2 samples were closely related to published sequences from the GenBank, such as a Connecticut isolate from 1997 (AF039930) or a Japan isolate from 2001 (AB189950). These analyses did not show any temporal distribution for either HPIV-1 or HPIV-2.

HPIV-3 showed no discernible groups, strains, or clades. Finally, the small sample size of available HPIV-4 samples limited our ability to perform a detailed phylogenetic analysis. However, the two samples collected in Peru were from the HPIV4a subtype and the one collected in Argentina corresponded to the HPIV4b subtype. All sequences are available on GenBank accession numbers #JQ268801 – JQ268898 and JQ905100 – JQ905102.

## Discussion

Our results emphasize that HPIV is a significant cause of respiratory morbidity in Latin America and should not be overlooked by doctors, clinical microbiologists, and public health officials. Unlike other common viral etiologies of respiratory disease—such as influenza virus, adenovirus, and RSV—neither an effective vaccine nor antiviral medication is available to combat HPIV. We show the presence of the four types of HPIV in Latin America, including HPIV-4, detected for the first time in our surveillance network during 2010. Others from the region have described slightly higher isolation rates of HPIV in ILI than our value of 3·2%; however, most of these studies focused on children. Examples of this are a study in Mexico between 2003 and 2004 that showed that 5% of the children with acute respiratory illness (ARI) had positive tests for HPIV-1 and HPIV-3,^15^ and a study from Brazil, published in 1991 that found that 16% of ARIs in ambulatory children were caused by HPIV-1, HPIV-2, and HPIV-3.^11^ The tropical city of Fortaleza in the Brazilian state of Ceará had a positivity rate of 3·8% in individuals under sixteen who attended the study's health facility with ARI.^13^ A study performed at the Vanderbilt Vaccine Evaluation Unit reported that 5·6% of viral cultures from children under 5 years of age with ILI and no underlying chronic disease were positive for this virus.^5^ In contrast to previous publications cited, our study enrolled participants of all ages, and we found that the odds of isolating HPIV-1, HPIV-2, or HPIV-3 in children 5 years or younger were more than four times higher than in those older than 5 years.

The most frequently isolated HPIV was type 3, an outcome widely described in this and other regions.^5^,^13^,^24^–^26^ However, the seasonal behavior of HPIV-3 and the other HPIVs varies from place to place, a finding attributed by some to climatic characteristics.^13^,^27^ Unfortunately, our data related to weather factors during the study period were limited, and we were not able to assess the association between weather and viral epidemiologic patterns.

Multiple studies in the region, such as those from Mexico 15 and Colombia,^14^ describe the presence and characteristics of HPIV-1 and HPIV-3; a few, such as one from Argentina,^3^ mention certain characteristics of HPIV-2, an HPIV type isolated in all of this study's cities. However, none of them describe the presence of HPIV-4,^12^,^13^,^15^,^28^ unlike our study, which found HPIV-4 in 5 of 10 participating countries. This type has been associated with milder respiratory disease 29,30 and a lower cell culture recovery rate,^30^ two reasons its prevalence in respiratory surveillance samples may have been missed. Nevertheless, despite the low pathogenic capability attributed to it, HPIV-4 has the capacity to trigger an outbreak.^31^

Although an initial screen with three cell lines was used for HPIV detection, direct PCR or hemadsorption assays would have further increased our HPIV identification rates, as previously suggested.^32^,^33^ Furthermore, initial screening with culture may have also impacted the identification of specific HPIV types and clades, as well as viruses other than HPIV, given that not all respiratory pathogens grow equally in culture media.^32^,^34^ Also, despite DFA staining performed 10 days (or 13 days in the case of Vero cells) in all specimens with no observed CPE, viral death may still have resulted in an underreporting of pathogen presence in some cases.

Nevertheless, we were able to isolate six other respiratory pathogens simultaneously with HPIV, with influenza virus A being the most frequent, although unlike all of the other respiratory viruses, we used both screening PCR and culture to identify influenza viruses.

Even though identifying two respiratory viruses in those with ILI is not rare, attributing specific clinical relevance to this finding remains challenging. Often, published reports associate a single pathogen with a particular clinical outcome. Nevertheless, of the two participants who developed multi-organ failure in our study, one had two respiratory viruses identified: HPIV-3 and herpes simplex virus. However, the percentage of those hospitalized with more than one virus (7·6%) nearly equaled the percentage of non-hospitalized participants with more than one respiratory virus identified (8·1%), indicating no link between the presence of multiple respiratory viruses and disease requiring hospitalization (Tables 1 and 3).

While some studies restricted their analyses to hospitalized patients,^28^,^35^ we considered hospitalization an outcome variable used to stratify severity of infection observed in the outpatient setting, although we were not able to be follow subjects as inpatients, a limitation of this study. As described by others, we found that those under 5 years old had higher hospitalization rates, probably because their respiratory and immune systems are still immature, predisposing to more severe respiratory infections.^36^ The type most frequently isolated in this young population and the overall hospitalized group was HPIV-3, consistent with other reports of severe manifestations (such as bronchiolitis and pneumonia) leading to hospitalization.^1^,^5^ The higher pathogenicity of HPIV-3 could be because it more readily infects apical and basolateral domains of A549 cells,^37^ human alveolar epithelial cells that diffuse substances such as water and electrolytes across the alveoli, and synthesize lecithin, involved in maintaining the membrane phospholipids of cells. Also, unlike other HPIVs, HPIV-3 has two receptor-binding sites for the hemagglutinin-neuraminidase protein, vital in activation of the fusion protein, which mediates fusion of the viral envelope with the host cell membrane. Nevertheless, we acknowledge that our findings of severe disease are most likely underestimates, given that surveillance was conducted mostly in clinics not co-located with an inpatient hospital, and individuals could have been hospitalized after this particular visit.

Upon examining total samples collected by month, the highest proportion of HPIV was observed between the months of June and August, although our findings did not have the power to establish seasonality as has been performed in the USA,^38^ considering seasonality as a characteristic of observed data in which regularity and predictability exist in defined time periods. In addition to raw numbers, we also plotted the proportion of total specimens yielding HPIV per month to control for transient factors that may have impacted total samples collected for a given region or time. As our sites differed vastly in climate, elevation, and latitude, we chose four locations that reflected this variation (Figure 2). The most significant finding in both Medellin, Colombia, and Managua, Nicaragua, was the increased proportion of HPIV-3 occurring March through May. Similar results for HPIV-3 have been reported in the USA 6 and Mexico.^15^ Our HPIV-3 results from Buenos Aires, Argentina, did not demonstrate this, and the proportions of HPIV-1 and HPIV-2 actually seemed to increase in the autumn season (mid-March through mid-June), which differs from the HPIV-1 findings of a US study.^38^

Piura, Peru, a site close to the Equator, did not show any discernible seasonality except for the described peaks, but did demonstrate all three HPIV types circulating at the same time. Others from Fortaleza, Brazil, located only 2 degrees north of Piura, found a significant seasonal pattern of HPIV-3 (*P* < 0·0001), occurring between September and November,^13^ their rainy season (while in Piura was during the dry period between June and July), indicating that seasonality may not solely be associated with distance from the Equator. Still, our amount of samples may have prevented us from detecting low-amplitude events that could have changed our results. Unlike the previous examples, most seasonal associations of HPIV were described in temperate areas,^6^,^39^,^40^ whereas studies in tropical regions such as Kenya,^25^ Taiwan,^26^ or Singapore 27 have failed to show seasonality of HPIV-1, HPIV-2, or HPIV-3. Despite the large number of samples obtained, we acknowledge that the amount of samples obtained and selected for phylogenetic characterization was not evenly distributed over all the countries and sites, a factor that may have affected our analysis.

During the 2009 pandemic, influenza A/H1N1 emerged in several regions of Latin America. Its presence did not have an impact on HPIV in the selected cities above the equator (Figure 2). On the other hand, the southern hemisphere cities of Piura and Buenos Aires had virtually no identifications of HPIV-1 and HPIV-2 during 2009, despite the increased number of ILI samples collected due to the pandemic. The impact of the pandemic influenza virus on the distribution of other respiratory viruses has also been described for H3N2.^41^

The phylogenetic analysis showed nucleotide variability but very little amino acid variability in all four HPIVs. Although isolates could be grouped into clades, more comparison sequences could provide information for a better classification. With our data, we could not identify any pattern or predominant HPIV group in any specific site or timeframe, as most of the samples were related to sequences collected in very different regions of the world, such as the USA, Canada, Saudi Arabia, or China.

Despite the prevalence of HPIV in our ILI study and others, antivirals and vaccines with proven effectiveness are lacking. Although a case report suggested efficacy of intravenous ribavirin combined with immunoglobulin,^42^ a larger study failed to show benefit with oral ribavirin against HPIV and other paramyxoviruses.^43^ A phase I study evaluating an HPIV-3/RSV vaccine demonstrated a favorable seroresponse and safety profile.^44^ As this vaccine and new antivirals continue to be evaluated, further phylogenetic, epidemiologic, and clinical studies may better delineate the approach and need for effective HPIV countermeasures.

## Addendum

Manuel V. Villaran (United States Naval Medical Research Unit No. 6, Lima, Peru) contributed to design of the study, writing of the article, and analysis and interpretation of all the data. Josefina Garcia (United States Naval Medical Research Unit No. 6, Lima, Peru) performed design of the article, analysis and interpretation of laboratory data, and revision of the intellectual content. Jorge Gomez (Direccion General de Epidemiologia, Ministerio de Salud, Lima, Peru) involved in design of the study and article, analysis and interpretation of Peruvian data, and revision of the intellectual content. Ana E. Arango (Universidad de Antioquia, Medellin, Colombia) and Marina Gonzales (Secretaria Seccional del Meta, Villavicencio, Colombia) contributed to design of the study and article, analysis and interpretation of Colombian data, and revision of the intellectual content. Wilson Chicaiza (Hospital Vozandes and Universidad de las Americas, Quito, Ecuador) and Washington Aleman (Clinica Alcivar and Hospital Vernaza, Guayaquil, Ecuador) involved in design of the study and article, analysis and interpretation of Ecuadorian data, and revision of the intellectual content. Ivette Lorenzana de Rivera (Universidad Nacional Autónoma de Honduras, Tegucigalpa, Honduras): contributed to design of the study and article, analysis and interpretation of data from Honduras, and revision of the intellectual content. Felix Sanchez (Hospital Infantil Manuel de Jesus Rivera, Managua, Nicaragua) performed design of the study and article, analysis and interpretation of Nicaraguan data, and revision of the intellectual content. Nicolas Aguayo (ONG rayos de Sol, Asuncion, Paraguay) contributed to design of the study and article, analysis and interpretation of Paraguayan data, and revision of the intellectual content. Tadeusz J. Kochel (United States Naval Medical Research Unit No. 6, Lima, Peru) involved in design of the study, analysis and interpretation of the data, and intellectual review of the article content. Eric S. Halsey (United States Naval Medical Research Unit No. 6, Lima, Peru) contributed to design of the study, analysis and interpretation of the data, and article writing and intellectual review of the content.
